# Water Stress and Black Cutworm Feeding Modulate Plant Response in Maize Colonized by *Metarhizium robertsii*

**DOI:** 10.3390/pathogens13070544

**Published:** 2024-06-27

**Authors:** Imtiaz Ahmad, Maria del Mar Jimenez-Gasco, Mary E. Barbercheck

**Affiliations:** 1 Department of Entomology, The Pennsylvania State University, University Park, PA 16802, USA; 2 Department of Plant Pathology and Environmental Microbiology, The Pennsylvania State University, University Park, PA 16802, USA; mxj22@psu.edu

**Keywords:** Clavicipitaceae, endophytic insect pathogenic fungus, *Metarhizium robertsii*, pest stress, plant defense gene expression, phytohormones, water stress

## Abstract

Plants face many environmental challenges and have evolved different strategies to defend against stress. One strategy is the establishment of mutualistic associations with endophytic microorganisms which contribute to plant defense and promote plant growth. The fungal entomopathogen *Metarhizium robertsii* is also an endophyte that can provide plant-protective and growth-promoting benefits to the host plant. We conducted a greenhouse experiment in which we imposed stress from deficit and excess soil moisture and feeding by larval black cutworm (BCW), *Agrotis ipsilon*, to maize plants that were either inoculated or not inoculated with *M. robertsii* (Mr). We evaluated plant growth and defense indicators to determine the effects of the interaction between Mr, maize, BCW feeding, and water stress. There was a significant effect of water treatment, but no effect of Mr treatment, on plant chlorophyl, height, and dry biomass. There was no effect of water or Mr treatment on damage caused by BCW feeding. There was a significant effect of water treatment, but not Mr treatment, on the expression of bx7 and rip2 genes and on foliar content of abscisic acid (ABA), 2,4-dihydroxy-7-methoxy-1,4-benzoxazin-3-one (DIMBOA), and gibberellin 19 (GA19), whereas GA53 was modulated by Mr treatment. Foliar content of GA19 and cis-Zeatin (cZ) was modulated by BCW feeding. In a redundancy analysis, plant phenology, plant nutrient content, and foliar DIMBOA and ABA content were most closely associated with water treatments. This study contributes toward understanding the sophisticated stress response signaling and endophytic mutualisms in crops.

## 1. Introduction

Environmental stressors such as excess or deficit soil moisture, excess salinity, pest feeding, and nutrient deficiency are major growth-limiting factors to crops in agricultural systems [1,2]. Plants have evolved mutualistic associations with naturally occurring soil microorganisms to defend against damage from these stressors. In associations with soil microbes, plant hosts can benefit through plant growth promotion [3], modulation of plant defenses [4], nutrient transfers [5], and suppression of plant disease [4,6] and herbivorous insect pests [7]. Among beneficial microbes, entomopathogenic fungi (EPF) in the genus *Metarhizium* Sorokin (Hypocreales: Clavicipitaceae), when associated with plants as endophytes, confer defensive and growth-promoting benefits to a wide range of plant species, including tomato [8], haricot bean [9], maize [3], wheat [10], cereal rye [11], Austrian winter pea [11], soybean [12], potato [13], cassava [14], and sweet pepper [15].

Plant hosts can avail a variety of benefits when endophytically colonized by *M. robertsii* J.F. Bisch., Rehner & Humber [16]. For example, tomato plants colonized by *M. anisopliae* had significantly greater root and shoot length and height compared with non-colonized control plants [8]. Many other studies have reported similar results with different host plants [3,4,11,12,13,17] and benefits to the host plant have been attributed to different mechanisms, including modulation of host plant molecular defense [4,18,19,20,21]. The jasmonic acid (JA) pathway in plants is directly related to biotic stress and is associated with response to infection by phytopathogens and feeding by herbivorous insects [22,23,24,25,26]. Studies reported that *M. robertsii*-colonized plants were primed to respond to environmental stressors epigenetically in the JA pathway and the expression of other plant defense genes was also modulated [4]. Other studies reported similar results with greater stress tolerance in *M. robertsii*-colonized plants, including salt stress and plant disease, compared to non-colonized plants [9,12]. Plants can better defend themselves from biotic stressors through such physiological responses [24,27]. The JA and abscisic acid (ABA) pathways have been shown to function synergistically where ABA production stimulates JA production, and vice versa [28,29].

In agricultural production, adverse weather conditions are common and a major obstacle to attaining high crop productivity and yield. Furthermore, stress from prolonged adverse weather, such as drought and flooding, and shifts in pests will be exacerbated due to changing climate conditions globally [30,31,32]. There are many gaps in scientific knowledge related to the endophytic lifestyle of *M. robertsii* and how different biotic and abiotic stressors affect endophyte–host plant interactions. Accurate assessment of the mutualism between plant hosts and *M. robertsii* will be improved by consideration of how they interact under biotic and abiotic stress [33].

In this study, we report the results of a greenhouse experiment to examine the interactions of endophytic colonization by *M. robertsii*, feeding by larval black cutworm (BCW), *Agrotis ipsilon* ((Hufnagel) (O. Lepidoptera: F. Noctuidae)), an economically important early-season pest of maize [34], and deficit, adequate, and excess soil moisture levels on the response of maize (*Zea mays* L.). We hypothesized that maize subjected to water stress (deficit or excess) and colonized by *M. robertsii* will have greater height, biomass, and chlorophyll content than non-colonized plants subjected to water stress. We also hypothesized that endophytically colonized maize exposed to the combined stress from deficit and excess water and feeding by BCW larvae will trigger plant defense response differently than non-colonized plants. To understand the interactions of endophytic colonization, BCW feeding, and water stress on maize response at the molecular level, we analyzed leaf samples from *M. robertsii*-colonized and non-colonized plants for gene expression and phytohormone content in different growth and defense-related pathways. We hypothesized that water stress and feeding by BCW will alter the expression of plant defense genes for pathways associated with defense against biotic and abiotic stress, and subsequently, there will be differential phytohormone content in *M. robertsii*-colonized and non-colonized maize.

## 2. Materials and Methods

### 2.1. Preparation of Inoculum of M. robertsii

We used an isolate of *M. robertsii* from a field experiment to determine the effects of cover crop species on the prevalence of *Metarhizium* spp. [4,35] that was stored on beads in cryovials at −80 °C (Pro-Lab Diagnostics Microbank™ Bacterial and Fungal Preservation System, Toronto, ON, Canada). To prepare inoculum for the experiment, we transferred beads containing spores of *M. robertsii* from cryovials to a Petri dish containing CTC medium [36] and incubated at 25 ± 2 °C in the dark for 14 days. Under aseptic conditions, the conidia were harvested and suspended in a sterile 0.05% aqueous solution (*v*/*v*) of Triton™ X-100 (Dow Chemical Co., Midland, MI, USA). We homogenized the conidial suspension by shaking it for one minute, then filtered the homogenized conidial suspension through four layers of sterile cheesecloth to separate the fungal mycelial fragments from conidia. To determine the concentration of the stock conidial suspension, we used a compound microscope at 400× magnification, estimated the conidia using a Neubauer hemocytometer, and adjusted the concentration to 1 × 10^8^ conidia mL^−1^ for use in experiments. To confirm the viability of the conidia, we plated 80 μL of the freshly prepared conidial suspension onto a Petri dish containing CTC medium and stored it in the dark at 25 ± 2 °C for 24 h. We assessed percent viability by randomly counting 200 conidia and considered conidia viable if the germ tube was at least twice the length of the conidium and only proceeded with the experiment when conidial suspensions had a germination rate of greater than 90%. Conidial viability was adequate in all replicates of the experiment.

### 2.2. Plant Growth Medium

We prepared a plant growth medium of field soil and potting mix (1:1 ratio (*v*/*v*); Vigoro Industries, Inc., Northbrook, IL, USA) using the methods described in Ahmad et al. [4]. To reduce the prevalence of other microbes, the growth medium was steamed twice for two hours at ~121 °C in a steam sterilizer [4]. One day after steaming, we filled steam-sterilized plastic pots (22.8 cm) with 5.5 L of steamed growth medium and placed the prepared pots randomly on a greenhouse floor with 16L:8D photoperiod at 25 ± 2 °C.

We surface-sterilized maize seeds (*Zea mays* var. 4050, Master’s Choice, organic) in a sterile laminar flow hood by immersion in 0.5% sodium hypochlorite for 2 min followed by soaking in 70% ethanol for 2 min and rinsing three times in sterile distilled water [37]. We confirmed the sterilization by plating the final rinsing water and three seeds separately onto Petri dishes, incubated in the dark at 25 ± 2 °C and monitored the plates for 7 days. We planted one dry sterilized seed into the prepared pots to a depth of ~2.5 cm in each pot using sterile spatulas. The plants were then grown with adequate water for 14 days before water stress treatments were imposed.

### 2.3. Insect Growth and Plant Damage

To impose feeding stress by black cutworm (BCW), a chewing insect pest of maize, we used 2nd instar BCW. We placed BCW eggs (Benzon Research Inc., Carlisle, PA, USA) on an artificial diet in an incubator at 27 °C and 16L:8D photoperiod [38]. After emergence, we weighed the larvae and transferred them to the whorls of 2-week-old plants using a fine paintbrush. We placed the plants in screen cages to prevent the escape of larvae, excluded other insects, and covered the base of the plants with aluminum foil to prevent larval contact with the soil. Larvae were allowed to feed for 5 days when they were removed and transferred into a pre-labeled, 30 mL clear plastic cup with a white paper insert lid (Frontier Agricultural Sciences, Newark, DE, USA) to measure their weight. We weighed the larvae using a digital balance and recorded their weights.

### 2.4. Treatment Groups and Experimental Plan

Following the initial 10-day period to allow maize germination and emergence, plants were divided into the following treatment groups: Treatment (1)—adequate moisture, no *M. robertsii*, no Triton X-100, no black cutworm; Treatment (2)—adequate moisture, +*M. robertsii,* +black cutworm; Treatment (3)—adequate moisture, no *M. robertsii*, +black cutworm; Treatment (4)—deficit moisture, +*M. robertsii*, +black cutworm; Treatment (5)—deficit moisture, no *M. robertsii*, +black cutworm; Treatment (6)—excess moisture, +*M. robersii*, +black cutworm; Treatment (7)—excess moisture, no *M. robertsii*, +black cutworm.

To inoculate pots in treatments with *M. robertsii*, we applied 10 mL of fresh conidia suspended in 0.05% aqueous solution (*v*/*v*) of Triton X-100 directly to the soil at the base of each test plant ten days after planting. Pots in treatments without inoculation of *M. robertsii* received 10 mL of 0.05% aqueous solution (*v*/*v*) of Triton X-100. For Treatment (1), we did not add Triton X-100 to use as a control.

We initiated water stress treatments 14 days after planting. We measured soil moisture level daily using an ML3 Thetaprobe (Dynamax Inc., Houston, TX, USA) soil moisture meter. We maintained soil moisture below 10% in the Deficit Water treatment, at 30–40% in the Excess Water treatment, and at 15–25% in the Adequate Water treatment. We maintained high soil moisture in the Excess Water treatment by placing a plastic pan under the pot to prevent water drainage and create waterlogging conditions.

We terminated the experiment by harvesting the plants at the 5th true leaf (V5). This corresponded to ~30 days after planting, ~16 days after the initiation of water stress and BCW inoculation, and ~20 days after inoculation with *M. robertsii*. We conducted three trials with ~10 plants in each treatment group in each trial, for a total of 206 plants.

### 2.5. Plant Responses

One day before terminating the experiment, we measured plant height and chlorophyll content. We measured plant height (cm) from the base of the plant to the tip of the longest fully emerged true leaf. We measured the total chlorophyll content of the fourth true leaf using a SPAD 502 Plus Chlorophyll Meter (Spectrum Technologies, Inc., Aurora, IL, USA) from three sections of the same leaf: the portion closest to the leaf node, the center of the leaf, and the portion near the tip of the leaf, and then used the mean of the readings. To avoid cross-contamination, we wiped the ruler and chlorophyll meter with 70% ethanol using Kimwipes™ between each plant. We measured above-ground biomass by cutting the plant at the soil–plant interface with clean scissors, excluding the fourth true leaf. We stored the maize biomass in dried, pre-weighed brown paper bags in the oven at 60 °C for 10–14 days when we weighed the biomass using a digital balance.

### 2.6. Plant Damage Rating

Simultaneously with measurements of plant height and chlorophyll content we assessed damage to maize caused by the BCW larvae. We assessed plant feeding damage by BCW with a rating scale (1 = no damage, 2 = little damage (pinholes), 3 = medium damage (10–25% of leaves damaged), 4 = heavy damage (50–75% of leaves damaged), 5 = entire plant damaged) as described by Toepfer et al. [39]. Although damage to maize by BCW was common in treatments receiving BCW, recovery of BCW larvae was very low. Therefore, we analyzed the plant damage rating and do not present data on the final BCW weights.

### 2.7. Confirmation of Endophytic Colonization

We evaluated the endophytic colonization of maize by *M. robertsii* at the end of the experiment (30 days after planting) [40]. From each plant, we removed the fourth true leaf and one 10 cm long primary root section. We rinsed the excised roots with tap water to remove soil. We surface-sterilized the excised leaf and root sections by submerging them in 0.5% sodium hypochlorite for three minutes followed by 70% ethanol for two minutes, followed by serially rinsing three times in sterile deionized water. We changed solutions between treatments to prevent contamination among the samples. We plated 80 μL of the final rinse water onto Sabouraud Dextrose Agar (SDA) medium and kept the dishes at 25 ± 2 °C for 10 days in darkness to confirm tissue sterilization.

Using sterile dissecting scissors, we cut off ∼1 mm of the outer edges of the surface-sterilized leaf and ends of the surface-sterilized root tissues to remove dead cells. We cut each leaf into six 6 × 6 mm sections and each root section into six 6 mm long sections so that each plant generated six leaf and six root sections. We plated each tissue type from each plant in a labeled Petri dish prepared with CTC medium by pressing the tissue flat against the surface of the medium. We sealed Petri dishes with parafilm and incubated them in the dark at 25 ± 2 °C for 14 days. We identified *M. robertsii* by its characteristic white hyphal growth and dark green conidia and confirmed its identity as *M. robertsii* by the methods of Kepler et al. [41].

We plated sections of leaf and root from 31, 32, and 28 plants from the Deficit, Adequate, and Excess Water treatments, respectively, from *M. robertsii*-inoculated treatments. We plated sections of leaf and root from 27, 60, and 28 plants, respectively, from the Deficit, Adequate, and Excess Water treatments from non-inoculated treatments. In total, we plated 546 root and 546 sections from 91 *M. robertsii*-inoculated plants and 690 root and 690 leaf sections from 115 control plants. We considered a plant to be endophytically colonized when we observed the growth of *M. robertsii* from one or more root or leaf sections. We calculated the proportion of endophytic colonization of plants among water treatments by dividing the total number of plants with root, leaf, or both root and leaf colonization within each water treatment group by the total number of *M. robertsii*-inoculated plants in that treatment. The intensity of colonization was calculated from the mean proportion of colonized tissue sections collected from *M. robertsii*-inoculated plants. Foliar colonization was rare; therefore, we present results for root colonization only.

### 2.8. Gene Expression Analysis and Phytohormone Profiles

We removed a 15 mm section of the fourth true leaf from each maize plant for analysis of defense gene expression and phytohormone content. We removed approximately 100–150 mg of the fourth true leaf and placed it into a pre-labeled 2 mL Eppendorf tube, flash froze the tube in liquid nitrogen, and then stored it at −80 °C until processing for gene expression and phytohormone content. To extract RNA from the frozen leaf tissue of control (non-inoculated) and *M. robertsii*-inoculated V5 maize plants, we homogenized 0.1 g of the leaf tissue in liquid nitrogen (GenoGrinder 2000, OPS Diagnostics, Lebanon, NJ, USA) followed by extraction with 1 mL of TRIzol (Life Technologies, Carlsbad, CA, USA) per ∼ 0.1 g of tissue. We quantified the genomic DNA-free RNA (Nanodrop, Thermo-Fisher Scientific, Waltham, MA, USA), and used 1 μg of total RNA to make complementary DNA (cDNA) (High-Capacity cDNA Reverse Transcription kit, Applied Biosystems, Waltham, MA, USA) and oligo(dT). Then, we performed qRT-PCR (7500 Fast Real-Time qPCR, Applied Biosystems, ThermoFisher Scientific, Inc.) with Fast Start Universal SYBR Green Master Mix (Roche Molecular Systems, Inc., Pleasanton, CA, USA) with actin as a reference gene and gene-specific primers (Supplementary Table S1). We used actin [42] as a reference gene and measured relative expression of the following genes: allene oxide synthase (aos) [43], 12-oxophytodienoate reductase 7 (bx7) [44], endochitinase A [45], lipoxygenase 1 (lox1) [46], maize protease inhibitor (mpi) [47], myeloblastosis (myb) [48], pathogenesis-related protein 5 (pr5) [49], plasma membrane intrinsic protein 1 (pip1) [50], tonoplast intrinsic protein 1 (tip1) [50], ribosome-inactivating protein 2 (rip2) [51], and WRKY transcription factor [52].

We conducted the qRT-PCR using the following default conditions: 50 °C for 2 min and 95 °C for 10 min; 95 °C for 30 s and 60 °C for 1 min repeated for 35 cycles; 72 °C for 10 min; and a dissociation stage. We performed RNA extraction, cDNA synthesis, and qRT-PCR for each biological replicate separately, with two biological replicates for each treatment and block (experiment replicate in time). In total, there were 36 biological replicates. There were three technical replicates, three endogenous controls, and three negative controls per biological replicate in each round of qRT-PCR.

Phytohormone profiling of pre-weighed maize leaf tissue was performed by the Proteomic and Metabolomic Facility of The Nebraska Center for Biotechnology at The University of Nebraska, Lincoln. The content of the following phytohormones was determined in each sample by HPLC/MS: abscisic acid (ABA), jasmonic acid (JA), jasmonoyl-isoleucine (JA-ILE), 12-oxophytodienoic acid (OPDA), salicylic acid (SA), 2,4-dihydroxy-7-methoxy-1,4-benzoxazin-3-one (DIMBOA), cis-Zeatin (cZ), cis-Zeatin riboside (cZR), gibberellin 19 (GA19), gibberellin 53 (GA53), indole-3-acetic acid (IAA), methylated indole-3-acetic acid (MethylIAA), and strigol. Here, we report results only for phytohormone content that differed significantly among experimental treatments.

### 2.9. Detection of M. robertsii in Soil

We used a sentinel insect bioassay method with larval *Galleria mellonella* as a bait to detect and provide a relative quantification of *M. robertsii* in soil at the end of the experiment [53]. A subsample of soil from each plant pot in all water and *M. robertsii* treatments was homogenized by hand and 250 mL were placed in a 473 mL plastic container along with 15, last-instar *G. mellonella*. Lids were placed on the containers, which were then stored at 20 °C for 10 days when insect condition was assessed and categorized as alive, dead from causes other than fungal infection, or infected by *M. robertsii*. Moribund and dead larvae exhibiting symptoms or signs of fungal infection were removed and rinsed briefly in 80% ethanol then in water and held in sealed humid chambers (59 mL Solo^®^ cups) with a small piece of moistened Whatman No. 1 filter paper for 7 days to observe the occurrence of characteristic green spores of *M. robertsii*. We confirmed the identity of *M. robertsii* by characteristic white hyphal growth and dark green conidia and confirmed identity as *M. robertsii* by molecular methods [41].

## 3. Statistical Analyses

We performed all statistical analyses in JMP^®^, Version 17 (SAS Institute Inc., Cary, NC, USA), unless stated otherwise. To determine the effect of water treatment, *M. robertsii*, and BCW on plant characteristics (height, biomass, chlorophyll content, root colonization frequency and intensity, and plant damage rating) we used univariate and multivariate statistical procedures. Foliar colonization was very rare with insufficient observations for statistical analysis. Therefore, we analyzed the data and present results based on root colonization by *M. robertsii*. We used mixed-model ANOVA to test whether the frequency (proportion of inoculated plants in which we detected *M. robertsii*) and intensity of root colonization (proportion of root sections per inoculated plant in which root colonization was detected) differed between water and *M. robertsii* treatments. The trial number (replicate in time) was considered an experimental block and coded as a random variable.

To identify soil variables with a significant effect on the variation in root colonization and response to treatments, we used forward selection multiple linear regression. The pool of explanatory environmental variables were soil chemical properties, determined by soil fertility analysis (Penn State Agricultural Analytical Services Laboratory (https://agsci.psu.edu/aasl/soil-testing/fertility (accessed on 24 June 2024)), included loss-on-ignition C [LOI-OM], K, Mg, P, Cu, Zn, Ca, S, cation exchange capacity [CEC], electrical conductivity [EC], pH, and gravimetric soil moisture, and the proportion of sentinel *G. mellonella* larvae infected by *M. robertsii* at the end of the experiment. We used the reduced set of explanatory variables to conduct a partial redundancy analysis (RDA) constrained by water and *M. robertsii* treatments with ‘CANOCO’ for Windows version 5.0 [54]. RDA results are displayed graphically with bi-plot scaling focused on standardized and centered inter-factor distances, where soil factors with a fit to the model of at least 20% are represented as solid line vectors.

Untransformed data are presented in the tables and figures. We considered the results of analyses significant at *p* < 0.05. For all analyses, we transformed proportions using square root arcsine transformation to meet assumptions of normality, equality of variances, and to reduce heterogeneity of variances.

## 4. Results

### 4.1. Endophytic Colonization of Maize by M. robertsii

#### 4.1.1. Mean Frequency of Detection in Inoculated Plants

We did not detect *M. robertsii* in plants in the treatments that were not inoculated with *M. robertsii*. There was no difference in the frequency of plant colonization among water treatments (*p* = 0.2488, df = 86.3, F_2,2_ = 1.41). The mean percentage of *M. robertsii*-inoculated plants in which endophytic colonization by *M. robertsii* was detected was 51.61% ± 9.12% (*n* = 31), 54.84% ± 9.08% (*n* = 31), and 34.48% ± 8.98% (*n* = 29) in the Deficit, Adequate, and Excess Water treatments, respectively.

#### 4.1.2. Mean Intensity of Colonization in Roots

There was no difference in the intensity of root colonization among water treatments (F = 1.59, df = 2, *p* = 0.2083). The mean intensity of root colonization was 40.53% ± 9.27%, 39.36% ± 9.28%, and 23.58% ± 9.47% of root sections colonized in the Deficit, Adequate, and Excess Water treatments, respectively.

### 4.2. Relationship between Water Stress, Endophytic Colonization, and Plant Growth

#### 4.2.1. Chlorophyll Content

There was a significant effect of water treatment (F = 14.07, df = 2, *p* < 0.0001) in which the chlorophyll content was lower in the Deficit (46.98 ± 1.35) compared to the Adequate (51.56 ± 1.36) and Excess (51.03 ± 1.36) treatments. The chlorophyll content of the fourth true leaf in the *M. robertsii* (Mr) (46.66 ± 1.47) and Triton X-100 (47.30 ± 1.52) treatments did not differ (F = 0.2375, df = 2, *p* = 0.6267). There was a significant interaction between the Mr and water treatments (F = 5.755, df = 2, *p* = 0.0039) in which chlorophyll content in Mr-inoculated plants in the Deficit and Excess Water treatments did not differ (*p* = 0.6557), while in the Triton X-100-treated plants, chlorophyll content was greater (*p* = 0.0002) in the Excess (53.44 ± 1.51) than in the Deficit Water (47.30 ± 1.52) treatment.

**Figure 1:**
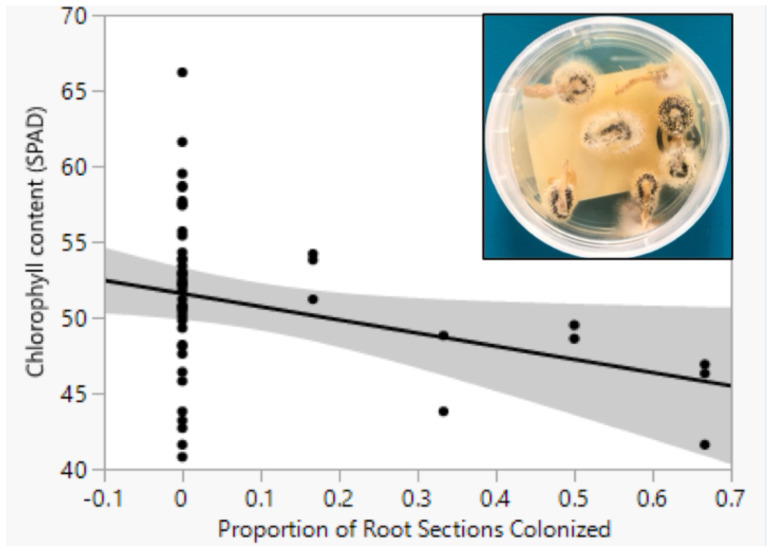
Relationship between chlorophyll content of the fourth true leaf of maize plants and intensity of root colonization by *M. robertsii*. Plant chlorophyll content was weakly (r^2^adj = 0.063) and negatively related to the intensity of root colonization by *M. robertsii*.

Within the Mr treatments, there was a weak but significant (*n* = 47, r^2^adj = 0.063, *p* = 0.0490) negative relationship between the intensity of root colonization and chlorophyll content in the Excess but not Adequate or Deficit Water treatments (Figure 1).

#### 4.2.2. Plant Height

Water (F = 23.78, df = 2, *p* < 0.0001), but not Mr (F = 0.1461, df = 1, *p* = 0.7025), treatment had a significant effect on plant height. Plants in the Deficit Water treatment were significantly shorter (78.0 ± 3.29 cm) than plants in the Adequate (97.33 ± 3.19 cm, *p* = 0.0002) and Excess (111.40 ± 3.33 cm, *p* < 0.0001) Water treatments. Plants in the Adequate Water treatment were significantly shorter (*p* = 0.0108) than in the Excess Water treatment. Plants in the Mr and Triton-X-100 treatments were 79.31 ± 4.56 cm and 76.69 ± 4.92 cm, respectively. There was no significant relationship between plant height and intensity of root colonization.

#### 4.2.3. Plant Dry Biomass

There was a significant effect of water (F = 31.78, df = 2, *p* < 0.0001) but not Mr treatment (F = 0.006, df = 1, *p* < 0.9387) on dry plant biomass. Plants in the Deficit Water treatment had significantly lower dry biomass (3.22 ± 0.534 gm) than plants in the Adequate (5.64 ± 0.529 gm, *p* = 0.0098) and Excess (9.83 ± 0.556 gm, *p* < 0.0001) Water treatments, and plants in the Adequate Water treatment had significantly lower dry biomass (*p* < 0.0001) than in the Excess Water treatment. The mean plant biomass in Mr and Triton X-100 treatments was 5.67 ± 0.416 gm and 6.79 ± 0.427 gm, respectively. There was no relationship between the intensity of root colonization and dry plant biomass.

#### 4.2.4. Plant Damage by Black Cutworm

There was no significant effect of water or Mr treatment on plant damage caused by BCW feeding. The mean damage rating, based on a rating system (1—no damage, 5—maximum damage) across treatments in which BCW was applied was 2.72 ± 0.09.

#### 4.2.5. Plant Foliar Nutrient Content

**Figure 2:**
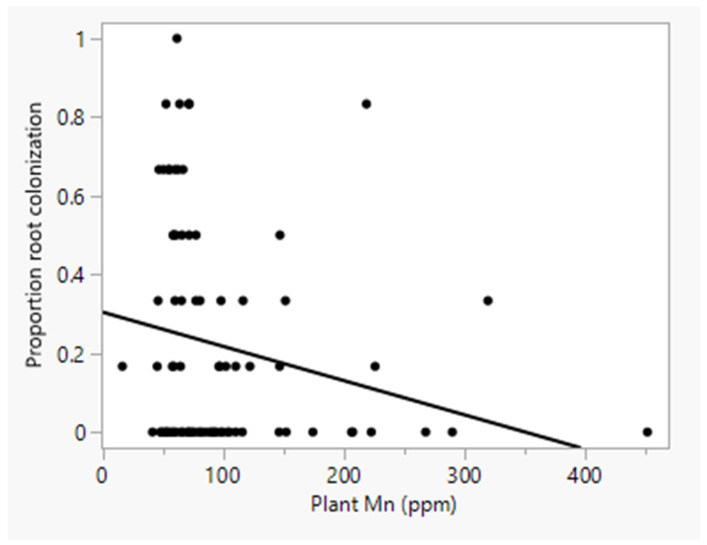
Relationship between plant Mn content and root colonization by *M. robertsii*. Plant Mn concentration was weakly (r^2^adj = 0.034) and negatively related to the intensity of root colonization by *M. robertsii*.

In forward selection stepwise multiple regression to determine the relationship between plant nutrient content and the intensity of root colonization by *M. robertsii*, only Mn content was significant across all water treatments. Plant Mn concentration was weakly and negatively related to the intensity of root colonization by *M. robertsii* (r^2^adj = 0.034, F = 4.1, *p* = 0.0459) (Figure 2). When the analysis was separated by water treatment, no plant nutrients were significantly related to the intensity of colonization.

### 4.3. Soil Nutrient Content

In forward selection stepwise multiple regression to determine the relationship between final soil nutrient content and the intensity of root colonization by *M. robertsii* across all water treatments, calcium (Ca, ppm), cation exchange capacity (CEC, meq/100 g), pH, and sulfur (S, ppm) were significant predictors for the intensity of root colonization (r^2^adj = 0.073, F = 2.773, *p* = 0.0320). Soil Ca was a positive predictor (*p* = 0.0130), while CEC (*p* = 0.0181), pH (*p* = 0.0250), and S (*p* = 0.0382) were negative predictors. In forward selection stepwise multiple regression to determine the relationship between soil nutrient content and the intensity of root colonization by *M. robertsii* within water treatment, none of the final soil fertility values were related to the intensity of root colonization.

### 4.4. Gene Expression

The expression of two genes of the ten analyzed showed differences due to water but not Mr treatment (Supplementary Table S2). There was a significant effect of water (F = 5.35, df = 2, *p* = 0.0165) but not Mr treatment (F = 0.182, df = 1, *p* = 0.6755) on the expression of bx7. Plants in the Excess Water treatment (2.85 ± 0.57) had a greater (*p* = 0.0128) expression of bx7 than in the Deficit Water treatment (0.89 ± 0.57). There was no difference in the expression of bx7 between plants in the Adequate (1.79 ± 0.47) and Excess (*p* = 0.1846) or Deficit (*p* = 0.3494) Water treatments. In an analysis within the Adequate Water treatment, there were no significant differences in bx7 expression between the Water-Only Control, Triton X-100 + BCW, and Mr + BCW treatments.

There was a significant effect of water (F = 8.59, df = 2, *p* = 0.0028) but not Mr treatments (F = 0.361, df = 1, *p* = 0.5555) on the expression of rip2. The expression of rip2 was greater in the Deficit Water (2.62 ± 0.30) treatment than in the Excess (0.92 ± 0.29, *p* = 0.0064) and Adequate (0.88 ± 0.29, *p* = 0.0053) Water treatments. There was no difference between the Excess and Adequate Water treatments.

### 4.5. Phytohormones

Among the twelve phytohormones analyzed, three were affected by water treatment, one was affected by Mr treatments, and two were modulated by BCW feeding. There was a significant effect of water treatment (F = 9.544, df = 2 *p* = 0.0006) but not Mr treatment (F = 0.178, df = 1, *p* = 0.6757) on ABA content. The ABA content in the Deficit Water treatment (142.42 ± 30.33 ng/g f.w.) was greater (*p* = 0.0017) than in the Adequate (24.16 ± 30.33 ng/g f.w.) and Excess (26.18 ± 30.33 ng/g f.w., *p* = 0.0020) Water treatments. There was no difference in the ABA content between Adequate and Excess Water treatments.

There was a significant effect of water treatment (F = 5.82, df = 2, *p* = 0.0068) but not Mr treatment (F = 0.13, df = 1, *p* = 0.72) on DIMBOA content. The DIMBOA content in the Deficit Water treatment (14,030.17 ± 1981.31 ng/g f.w.) was greater (*p* = 0.0048) than in the Excess Water (2641.82 ± 1981.31 ng/g f.w.) treatment. There was no difference in the DIMBOA content between the Adequate (8006.93 ± 1981.31 ng/g f.w.) and Excess Water treatments (*p* = 0.2569) or between the Adequate and Deficit Water treatments (*p* = 0.1839). There was no difference between the Mr (8741.18 ± 1636.43 ng/g f.w.) and Triton X-100 (7711.42 ± 1636.43 ng/g f.w.) treatments (F = 0.1074, df = 1, *p* = 0.7451).

There was a significant effect of water treatment (F = 5.71, df = 2, *p* = 0.0083) but not Mr treatment (F = 0.13, df = 1, *p* = 0.72) on GA19 content. The GA19 content in the Adequate Water (13.46 ± 1.37 ng/g f.w.) treatment was greater than in the Deficit Water (7.19 ± 1.37 ng/g f.w.) treatment (*p* = 0.008). There was no difference in the GA19 content between Adequate and Excess Water (8.72 ± 1.37 ng/g f.w.) treatments (*p* = 0.05) and between Excess and Deficient Water treatments (*p* = 0.71). There was no difference between the Mr (10.62 ± 1.2 ng/g f.w.) and Triton X-100 (8.96 ± 1.2 ng/g f.w.) treatments (F = 0.8614, df = 1, *p* = 0.3603).

In analyses to examine the effect of BCW feeding within the Adequate Water treatment, the non-inoculated control (Water-Only) differed from the Mr and Triton X-100 treatments, both of which included the addition of BCW (F = 8.02, df = 2, *p* = 0.005) on GA19 content. The GA19 content in the Water-Only Control treatment (32.27 ± 4.39 ng/g f.w.) was greater than in the Triton X-100 (13.9 ± 4.39 ng/g f.w., *p* = 0.0123) and Mr (13.01 ± 4.39 ng/g f.w., *p* = 0.0095) treatments. There was no difference in GA19 content between the Mr and Triton X-100 (*p* = 0.99) treatments.

There was a significant effect of Mr (F = 5.77, df = 1, *p* = 0.0232) treatment but not water (F = 2.37, df = 2, *p* = 0.1117) treatment on GA53. The concentration of GA53 was greater in the Mr + BCW (2.04 ± 0.25 ng/g f.w.) than in the Triton X-100 + BCW (0.94 ± 0.25 ng/g f.w.) treatment. Within the Adequate Water treatment, GA53 in the Water-Only Control (4.46 ± 0.78 ng/g f.w.) was marginally non-significantly greater (*p* = 0.0577) than in the Triton X-100 + BCW (1.44 ± 0.78 ng/g f.w.) treatment, and not different from the Mr + BCW (2.81 ± 0.78 ng/g f.w) treatment. The concentrations of GA53 in the Triton X-100 + BCW and Mr + BCW treatments were not different.

In analyses to examine the effect of BCW feeding within the Adequate Water treatment, treatment had a significant effect on cZ content (F = 6.59, df = 2, *p* = 0.0106). The cZ content in the Triton X-100 + BCW (3.39 ± 0.64 ng/g f.w.) treatment was greater than in the Water-Only Control treatment (0.47 ± 0.64 ng/g f.w., *p* = 0.0093). There were no differences in cZ content between the Mr + BCW (1.36 ± 0.64 ng/g f.w.) and Triton X-100 + BCW (*p* = 0.0714) treatments or the Water-Only Control treatment (*p* = 0.5266).

### 4.6. Multivariate Associations

Association of Plant Characteristics and Soil Nutrients

**Figure 3:**
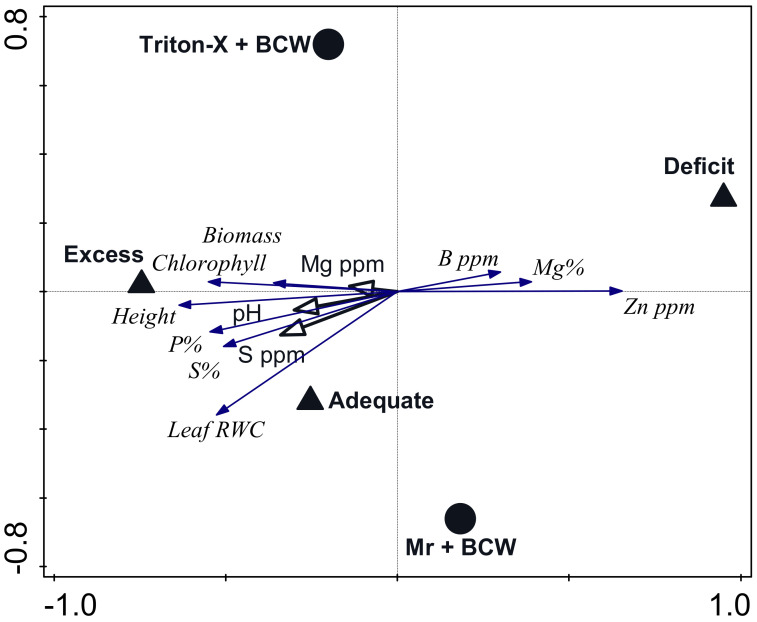
Biplot of a redundancy analysis with axes constrained to water + BCW (solid triangles) and Mr + BCW (solid circles) treatments. Axis 1 explained 11.83% of the variation and axis 2 explained 8.76% of the variation. Plant phenology measures and nutrient content are shown in italics with closed-arrow vectors. Supplementary soil variables are shown with open-arrow vectors. Only variables with at least 20% fit to the axes are shown. RWC = relative water content.

A redundancy analysis constraining Mr + BCW and water + BCW treatments and including end-of-experiment plant phenology and nutrient content as response variables and soil nutrient values as supplementary variables was significant for the first axis (pseudo-F = 7.0, *p* = 0.001) and all axes (pseudo-F = 18.1, *p* = 0.001) (Figure 3). The explanatory variables accounted for 24.3% of the observed variation (adjusted). The first axis, represented by water + BCW treatments, explained 11.83% of the variation, and axis 2, represented by Mr + BCW treatments, explained 8.76% of the variation. Plant phenology (height, biomass, chlorophyll content, leaf relative water content) and plant nutrient content (P, S, B, Mg, Zn) were most closely associated with water treatment (Figure 3). Supplementary soil variables with at least 20% fit to the model included pH, S, and Mg.

### 4.7. Gene Expression

A redundancy analysis constraining Mr + BCW and water treatments and including end-of-experiment relative quantification of plant defense gene expression as response variables was not significant for the first axis (pseudo-F = 1., *p* = 0.183) or all axes (pseudo-F = 1.9, *p* = 0.062).

### 4.8. Association of Plant Phytohormones with Treatments

**Figure 4:**
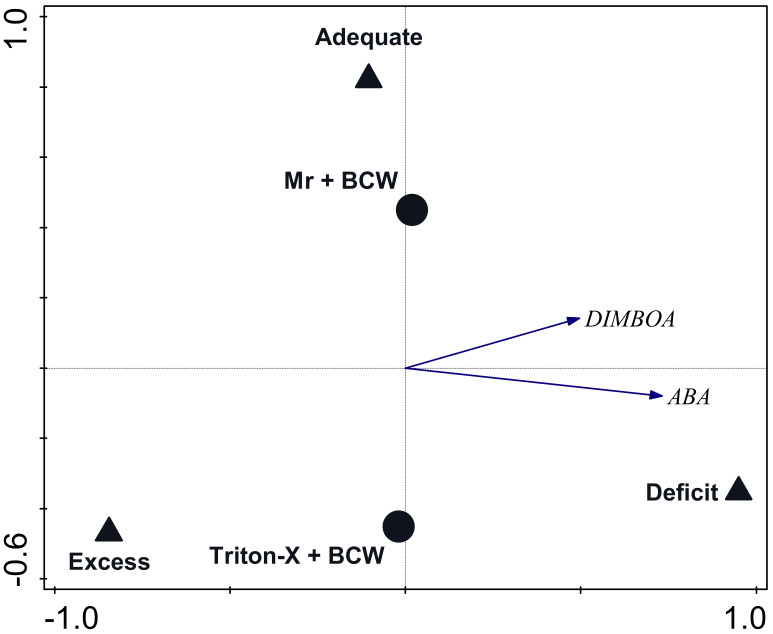
Biplot of a redundancy analysis with axes constrained to water (solid triangles) and Mr treatments (solid circles). Axis 1 explained 18.8% of the explained variation and axis 2 explained 5.9% of the explained variation. Plant phytohormone measures are shown in italics with closed-arrow vectors. Supplementary soil variables are shown with open-arrow vectors. Only variables with at least 20% fit to the axes are shown. Mr = *M. robertsii*; BCW = black cutworm, ABA = abscisic acid.

A redundancy analysis constraining Mr + BCW and water treatments that included end-of-experiment plant phytohormone content as response variables was significant for the first axis (pseudo-F = 2.5, *p* = 0.001) and all axes (pseudo-F = 3.6, *p* = 0.001). The explanatory variables accounted for 24.3% of the explained variation (adjusted). The first axis, represented by water treatment, explained 18.8% of the variation, and axis 2, represented by Mr + BCW treatment, explained 5.9% of the variation. The DIMBOA and ABA content were most closely associated with the Deficit Water treatment (Figure 4).

**Figure 5:**
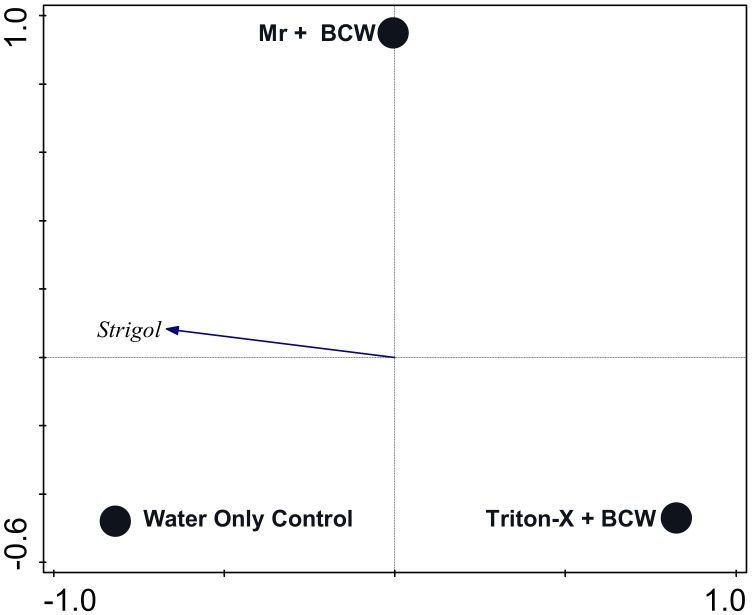
Biplot of a redundancy analysis with axes constrained to treatments within the Adequate Water treatment. Axis 1 explained 23.9% of the explained variation and axis 2 explained 3.7% of the explained variation. Plant phytohormone measures are shown in italics with closed-arrow vectors. Supplementary soil variables are shown with open-arrow vectors. Only variables with at least 20% fit to the axes are shown. Mr = *M. robertsii*; BCW = black cutworm.

A redundancy analysis constraining Mr + BCW and Triton X-100 + BCW treatments and the Water-Only Control treatment within the Adequate Water treatment that included end-of-experiment plant phytohormone contents as response variables was significant for the first axis (pseudo-F = 2.4, *p* = 0.023) and all axes (pseudo-F = 2.9, *p* = 0.02). The explanatory variables accounted for 18.1% of the variation (adjusted). The first axis, represented by the two treatments without *M. robertsii*, explained 23.9% of the explained variation, and axis 2, represented by Mr + BCW treatment, explained 3.7% of the variation. Strigol content was most closely associated with the Water-Only Control treatment (Figure 5).

## 5. Discussion

### 5.1. Plant Growth Response to Water Stress and Endophytic Colonization

Overall, plant responses to experimental treatments were associated with water stress as opposed to herbivory by black cutworm or colonization by *M. robertsii*. Among all test plants, there was no difference in the frequency or the intensity of plant colonization by *M. robertsii* (Mr) among water treatments. There was a significant effect of water treatment but not Mr treatment on plant growth parameters, including chlorophyll content, height, and aboveground biomass. The results of this research are not consistent with our previous study which demonstrated a positive effect of *M. robertsii* colonization on plant growth, including aboveground biomass and height in maize plants that were not subjected to water stress [4]. This difference may be due to the different inoculation methods. We used a soil inoculation method in this study compared with the seed inoculation method used in Ahmad et al. [4]. In other studies, endophytic root colonization by *M. robertsii*, *M. brunneum*, and *M. anisopliae* increased the stalk length, leaf collar formation, ear, and foliage biomass of maize [3]. In tomato, endophytic root colonization by *M. anisopliae* increased plant height, root length, and root and shoot dry weight [8].

Within the Mr treatments, there was a weak but significant negative relationship between the intensity of root colonization and chlorophyll content in the Excess but not Adequate or Deficit Water treatments. This negative correlation could be due to the hypoxic root environment that may have resulted from flooding conditions leading to lower chlorophyll content in the Excess Water treatment [55,56]. This could have led to overall no significant effect of endophytic colonization on plant growth parameters in the presence of water stress. A lack of effect of Mr treatment on chlorophyll content is consistent with previous results [3,4,14]. Analyzing the effects of water treatments on plant responses provided additional insights on the relationship between endophytic colonization, soil moisture, and maize growth, and it demonstrates the effect of context dependency on mediating plant–microbe interactions [20]. In mutualistic interactions, context dependency is the magnitude of change in interaction as the net positive or negative outcome is dependent on both the host and endophyte species, and their biotic and abiotic environments [57,58,59].

In our study, plant manganese (Mn) concentration was weakly and negatively related to the intensity of root colonization. When the analysis was separated by water treatment, no plant nutrient factors were significantly related to the intensity of colonization. We are unaware of other studies reporting a relationship between endophytic fungi and plant or soil Mn concentrations. Mn is an essential constituent during oxygenic photosynthesis, but at high concentrations, it restricts plant growth [60]. Some fungi and bacteria reduce the toxicity of Mn by oxidation [61], and Tsuji et al. [62] suggested that the production of biogenic Mn oxides in biofilms by some epiphytic or endophytic bacteria can elevate Mn levels inside plants. The negative relationship observed between plant Mn and intensity of colonization by *M. robertsii* in our study suggests some level of inhibition of *M. robertsii* by Mn, perhaps through indirect interactions with microorganisms tolerant to or capable of oxidizing Mn in soil, but this remains to be tested.

In analyses to determine the associations between soil nutrient content on the intensity of root colonization by *M. robertsii* across all water treatments, ppm calcium, cation exchange capacity (CEC, meq/100 g), pH, and ppm sulfur (S) were significant predictors for the intensity of root colonization. Soil Ca was a positive predictor, while CEC, pH, and S were negative predictors. To determine the effects of soil nutrient content on the intensity of root colonization by *M. robertsii* within water treatment, none of the final soil fertility values were related to the intensity of root colonization. Other studies have reported that the effects of endophytic *Metarhizium* spp. on host plants can be context-dependent depending on environmental conditions. Our observations are consistent with an earlier field study [35] in which soil calcium concentration was a positive predictor and CEC was a negative predictor for the prevalence of *M. robertsii* in soil. Sulfur is a common fungicide used to manage fungal plant diseases, inhibiting fungal growth due to its function as a strong oxidant [63,64]. The negative relationship between soil S concentration and intensity of colonization by *M. robertsii* is consistent with these reports.

### 5.2. Changes in Plant Defense Gene Expression

Water stress and herbivory by insects can lead to the induction of physical and chemical defenses that promote plant fitness [56], as well as a reduction of major cell processes involved in growth and photosynthesis [65]. Several phytohormones function in regulating plant defense, including jasmonic acid (JA), salicylic acid (SA), abscisic acid (ABA), ethylene, auxin, and cytokinins [66,67,68,69]. Maize plants respond to stressors by triggering defense signaling, changes in gene expression, and biosynthesis of specialized metabolites [70]. Benzoxazinoids (BXs) are secondary plant metabolites in grasses, including economically important crops such as maize, wheat, and rye [71] with a high potential for chemical defense against biotic and abiotic stresses, including herbivory, phytopathogens, and drought [72]. Niculaes et al. [73] provided a comprehensive overview of the biosynthesis, metabolism, and biological activities of benzoxazinoids. Benzoxazinoids are synthesized in seedlings and stored as glucosides [44]. Bx7 is a gene involved in the biosynthesis pathway of BXs. Specifically, bx7 catalyzes the conversion of TRIMBOA-Glc to DIM2BOA-Glc by O-methyltransferase. Leaf metabolites in maize are very sensitive to drought, and Zhang et al. [74] found that benzoxazinoid genes, especially bx12, were upregulated in deficit compared to well-watered conditions. In our study, there was a significant effect of water but not Mr treatment on the expression of bx7. Plants in the Excess Water treatment had a greater expression of bx7 than in the Deficit Water treatment and there was no difference in the expression of bx7 between Excess and Adequate Water treatments. We suggest that a higher expression of bx7 in Excess and Adequate Water treatments in comparison to the Deficit Water treatment may reflect the greater ability of the plant to mount a defense response in the relative absence of water stress, or that other Bx genes, such as bx12, may be involved in responding to Deficit Water stress in the maize variety used in our assays. The greater foliar DIMBOA concentrations in plants in the Deficit Water treatments compared with the Excess and Adequate Water treatments in our study indicate that this may be the case. Comprehensive metabolomic studies may be needed to better understand the response of maize to combined biotic and abiotic stresses.

Some plant-derived toxic proteins, such as ribosome-inactivating proteins (RIPs), function in defense against viruses, pathogens, and insects [75,76,77]. RIPs are cytotoxic enzymes that inhibit protein translation by depurinating ribosomal RNA [78]. RIPs are involved in the jasmonate biosynthesis pathway that helps to regulate the production of toxic metabolites and a wide variety of other responses to insect herbivory [79,80,81]. These anti-herbivore defensive proteins are induced by insect feeding to deter insect feeding and may regulate general plant defense [51]. The toxin rip2 is also induced by caterpillar feeding and is one of a potential suite of proteins that defend maize against chewing herbivores [51]. In this study, we found a significant difference in the expression of rip2 among water, but not Mr, treatments. Plants in the Deficit Treatment had a greater expression of rip2 than in the Adequate and Excess treatments, which did not differ. We suggest that a higher expression of rip2 in the Deficit Water treatment may be a defense strategy to protect plants under drought conditions, due to its involvement in the biosynthesis of jasmonates that may act synergistically with ABA and provide protection against abiotic as well as biotic challenges.

### 5.3. Phytohormone Content Modulation in Reponse to Stress and Endophytic Colonization

Phytohormones are associated with the modulation of plant growth and defense [82]. Plant–fungal symbioses may result in modulation of the defense signaling cascade as an alternative adaptive strategy to cope with hostile environments. Several phytohormones, such as gibberellins, ABA, auxins, and cytokinins can modulate water stress tolerance in plants [82,83]. In this study, we did not detect significant changes in the pattern of phytohormone content in maize plants due to endophytic colonization by *M. robertsii*. However, we found some significant interactions between water and Mr treatments. In this study, the foliar content of five phytohormones, ABA, DIMBOA, GA19, GA53, and cZ, were significantly affected by water treatment.

ABA is associated with the modulation of plant growth and defense against abiotic stress, including water stress [84,85]. In water-deficit conditions, ABA initiates water-saving activities such as stomatal closure, increased root growth, and reduced leaf expansion [86]. ABA content was significantly greater in plants in the Deficit Water treatment compared to the Adequate and Excess Water treatments. We suggest that crosstalk between gene transcription and ABA synthesis intensified the response of plants to drought, resulting in significantly greater ABA concentration in plants in the Deficit Water treatments. These results are consistent with our earlier study that reported greater foliar concentrations of ABA in deficit water-stressed maize [20].

Greater ABA concentrations also may have resulted in a higher level of rip2 gene transcription in the Deficit Water treatment compared with Excess and Adequate Water treatments. ABA interacts synergistically and antagonistically with other phytohormones such as JA [87,88]. ABA and JA share multiple signaling genes which regulate the expression of major signaling genes in the JA pathway [28,29].

DIMBOA is an anti-insect benzoxazinoid compound primarily associated with defense against herbivorous insects [89]. However, DIMBOA synthesis has been demonstrated to be stimulated by increased concentrations of ABA and JA [88,90]. In the Deficit Water treatments, DIMBOA concentration was significantly greater than in the Excess Water treatment. Belowground stimulation of ABA content, triggered by drought conditions, has been demonstrated to increase aboveground concentrations of DIMBOA [90]. We suggest that the increase in DIMBOA concentration that we observed in plants in the Deficit Water treatments was associated with increased levels of ABA. Generally, phytohormones can interact synergistically and antagonistically with one another, but context dependency does not help to predict whether certain phytohormones will always interact in the same way without exception in different plant species [70,91].

Gibberellins (GA) are primarily involved in plant growth regulation and development [92], but recent reports revealed that they also regulate certain biological processes in response to stress [93]. GA and ABA antagonistically mediate many plant-developmental processes [94]. In the Adequate Water treatments in our study, GA19 content was lower in plants with black cutwork feeding compared with the Water-Only Control treatment. However, the concentration of GA53 was greater in the Mr + BCW than in the Triton X + BCW treatment. Within the Adequate Water treatment, GA53 in the Water-Only Control was marginally but non-significantly greater than in the Triton X + BCW and not different from the Mr + BCW treatment. We suggest that the modulation of GA19 and GA53 observed with black cutworm feeding may be due to the activation of other defense pathways by herbivory. GA may have acted antagonistically to ABA to regulate plant growth and defense under limiting conditions. Some endophytes produce GA and auxins in planta that promote plant growth [12,95,96]. Hu and Bidochka reported that combined or separate inoculation with either *M. robertsii* or the phytopathogenic *F. solani* did not induce any changes in GA content in common bean leaf tissue compared with non-inoculated control plants [97].

cZ (cis-Zeatin) is a form of cytokinin, a phytohormone primarily associated with plant growth and development in seedlings and immature plants, particularly in growth-limiting conditions [98,99]. cZ was upregulated in the presence of increased JA concentration, linking it to biotic stressors [99]. Additionally, the concentration of cZ increased dramatically in plants with drought conditions [98]. Our results are consistent with these studies, where cZ concentration was significantly greater in plants with black cutwork feeding compared to no-feeding control plants. We suggest that concentrations of cZ were significantly upregulated due to crosstalk with the JA pathway that may have been modulated due to black cutworm feeding. Maize generally has very high levels of cZ, which suggests that these bioactive molecules are not used only under stress or growth-limiting conditions but are also present at the basal level [100].

Different forms of JA activate plant defense responses to biotic stressors, such as attack by phytopathogens and herbivorous insects [4,24] as well as by abiotic stressors such as salinity [101], drought [102,103], and UV irradiation [104]. Previous studies have reported that endophytic colonization by *Metarhizium* spp. increases JA concentration in host plants [4,12]. We suggest that the central role of JA as a stress regulator in plants and crosstalk with the ABA pathway influenced the effect of water treatment on JA concentration. The difference in phytohormone response to water treatments demonstrates how the JA pathway functions differently under different environmental conditions, which may be in part due to the complex negative and positive relationships between JA and other phytohormone pathways [21,67,70,103,105].

## 6. Conclusions

Our study provides insight on plant defense and growth regulation by the interaction of stress response and endophytic colonization by the mutualist insect-pathogenic fungi, *M. robertsii*. It sheds light on how maize shows a sophisticated defense and growth response when it faces multiple challenges in the ecological setting.

Several questions remain to be explored before endophytic insect pathogens, such as *Metarhizium* spp., can be predictably exploited in the field for plant stress management. For example, information critical for the deployment of this approach, such as the variability of response of endophytes and endophyte-colonized plants species and crop varieties to diverse biotic and abiotic stresses, ecological competency among species and isolates of *Metarhizium* in agricultural soils, the prevalence and persistence of natural and managed endophytic colonization, and mechanisms of action of plant-growth promoting and pest suppressive effects of endophytic *Metarhizium* spp. remain to be better understood.

## Data Availability

The data presented in this study are available on request from the corresponding authors.

## Supplementary Materials

The following supporting information can be downloaded at: https://www.mdpi.com/article/10.3390/pathogens13070544/s1, Figure S1. Relationship of plant height, biomass, chlorophyll content, intensity of root colonization (Mr intensity), and relative prevalence of M. robertsii (Mr prevalence) in the soil at the end of the experiment with Water (Deficit, Adequate, Excess) and Mr (M. robertsii, Triton X-100), treatments; Figure S2. Relationship of plant growth parameters and relative prevalence of M. robertsii in the soil at the end of the experiment; Figure S3. Metarhizium robertsii growth on potato dextrose agar (PDA) supplemented with yeast at 25 ± 2 °C for 10 days in darkness; Figure S4. Plant tissues show growth of M. robertsii when plated on CTC, a selective medium that confirms endophytic colonization in maize; Figure S5. Mealworm larvae covered with dark green conidia of Metarhizium robertsii. Table S1. Forward and reverse sequences of gene primers tested in this study and actin (endogenous control); Table S2. Mean (±st. error) of the relative quantification of the expression of defense-related genes from maize foliage in the Adequate Water treatments.
