# Supplementary material for: Water Stress and Black Cutworm Feeding Modulate Plant Response in Maize Colonized by Metarhizium robertsii
Source: Pathogens. 2024 Jun 27;13(7):544. doi: 10.3390/pathogens13070544 (PMC11280422; doi:10.3390/pathogens13070544)
Supplement: Supplementary file 1 [file pathogens-13-00544-s001.zip › pathogens-3010937-supplementary.pdf]

Supplementary Data

Table S1. Forward and reverse sequences of gene primers tested in this study and actin (endogenous control).

Gene	Forward Primer (5′-3′)	Reverse Primer (5′-3′)	Reference
Actin	GGAGCTCGAGAATGCCAAGAGCAG	GGAGCTCGAGAATGCCAAGAGCAG	[42]
Allene oxide synthase (*aos*)	CAAACCGACGAATTTGAGCAA	GGAGGCTCGCAACAAGTTG	[43]
12-oxophytodienoate reductase 7 (*bx7*)	TCGACCGCTGCGGCGGGAGC	ATGCTCTCGTAGAAGCTGGCCCT	[44]
Endochitinase A	CAAGACGGCGCTCTGGTT	AAGCCCTGCGGCATCA	[45]
Lipoxygenase 1 (*lox1*)	CGTTCCGTGAAGTGTGGTTCT	CGTTCCGTGAAGTGTGGTTCT	[46]
Maize protease inhibitor (*mpi*)	GCGGATTATCGCCCTAACC	CGTCTGGGCGACGATGTC	[47]
Myeloblastosis (*myb*)	GTCCGTGACAAGGACCAAGAA	ACCCCAACAGGATCAGGTGTT	[48]
Pathogenesis-related protein 5 (*pr5*)	GTCATCGACGGCTACAACCT	GGGCAGAAGGTGACTTGGTA	[49]
Plasma membrane intrinsic protein 1 (*pip1*)	GCGCCGCCGTAATTTACA	GCCGACCCAGAAGATCCA	[50]
Ribosome inactivating protein (*rip2*)	GAGATCCCCGACATGAAGGA	CTGCGCTGCTGCGTTTT	[51]
WRKY transcription factor	GCTCGTCACCTACACCTTCG	AGCTTTCGTCCTCCTCTGC	[52]

Table S2. Mean (± st. error) of the relative quantification of the expression of defense-related genes from maize foliage in the Adequate Water treatments.

Defense-related Gene	Untreated control	Triton X-100 + BCW	*M. robertsii* + BCW
Allene Oxide Synthase (*aos*)	2.893 ±1.303	3.196 ± 2.214	6.761 ± 2.787
Endochitinase A	7.734 ± 3.779	69.375 ± 56.945	64.300 ± 61.622
Lipoxygenase 1 (*lox1*)	0.883 ± 0.170	1.485 ± 0.638	1.98 ± 0.081
Maize Protease Inhibitor (*mpi*)	1.811 ± 0.308	12.034 ± 9.541	2.813 ± 0.945
Myeloblastosis Gene (*myb*)	0.934 ± 0.0355	1.0195 ± 0.297	1.981 ± 0.946
12-oxophytodienoate Reductase 7 (*bx7*)	0.865 ± 0.139	1.369 ± 0.211	1.396 ± 0.279
Plasma membrane Intrinsic Protein 1 (*pip1*)	0.759 ± 0.118	1.691 ± 0.706	1.12 ± 0.340
Pathogenesis-related Gene 5 (*pr5*)	12.602 ± 11.493	2.298 ± 0.602	38.039 ± 35.330
Ribosome Inactivating Protein 2 (*rip2*)	0.570 ± 0.228	0.82 ± 0.235	1.011 ± 0.254
Tonoplast Intrinsic Protein 1 (*tip1*)	0.839 ± 0.172	2.135 ± 1.151	5.225 ± 2.949
WRKY Transcription Factor	0.289 ± 0.239	0.045 ± 0.008	0.044 ± 0.011

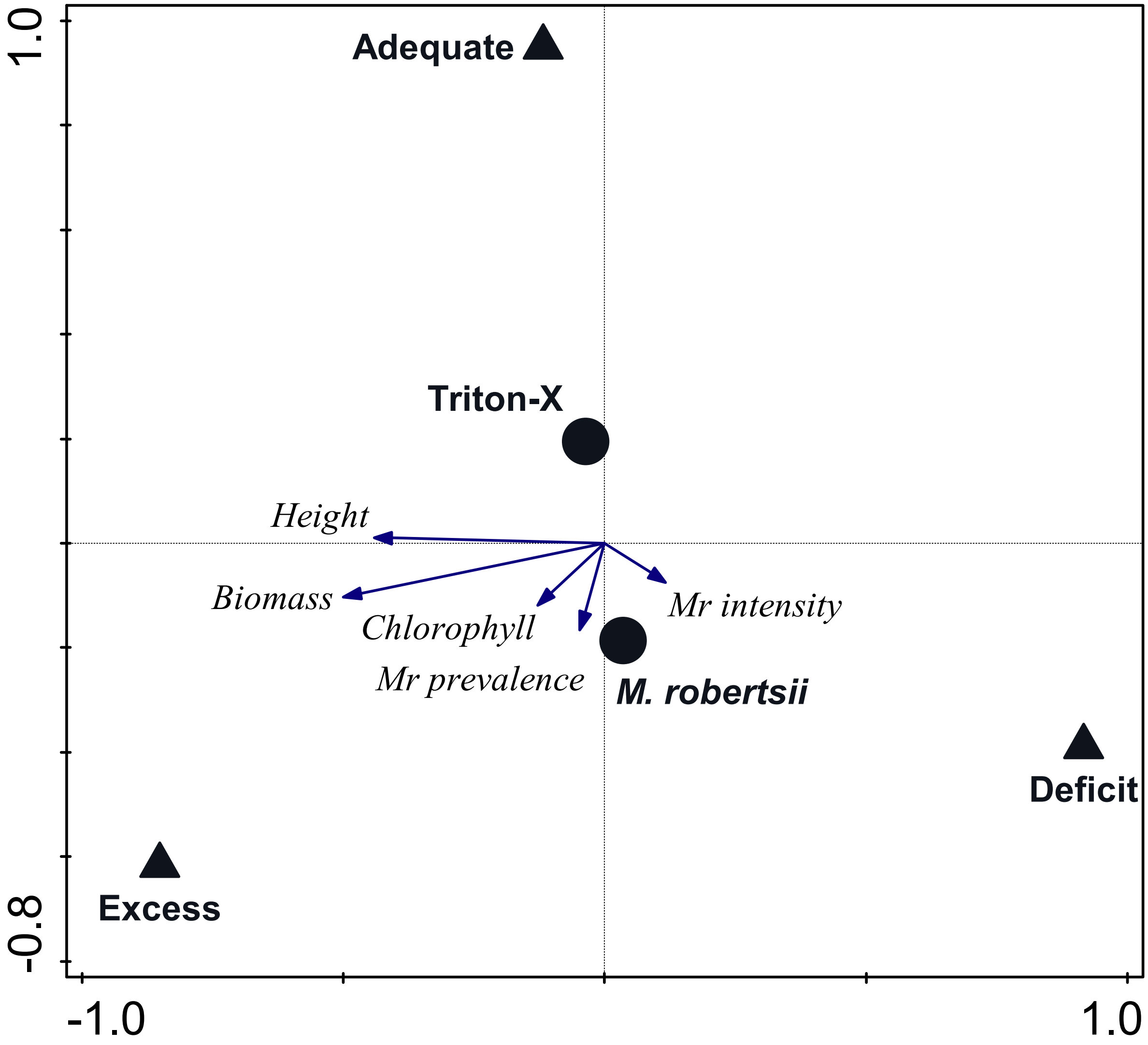

Figure S1. Relationship of plant height, biomass, chlorophyll content, intensity of root colonization (*Mr intensity*), and relative prevalence of *M. robertsii* (*Mr prevalence*) in the soil at the end of the experiment with Water (Deficit, Adequate, Excess) and Mr (*M. robertsii*, Triton X-100), treatments. Axis 1 explains 16.54% of the explained variation and Axis 2 explains 0.28% of the explained variation.

Association of plant characteristics with Water and Mr treatments

In redundancy analysis of plant responses constrained by Water and Mr treatments, explanatory variables accounted for 16.85% of the total variation. Water treatment (Axis 1) explained about 16.54% of the explained variation in plant characteristics. Mr treatment (Axis 2) only explained about 0.28% of the variation. Plant height and biomass, and weakly chlorophyll content, and the relative prevalence of *M. robertsii* in the soil at the end of the experiment were most closely related to the Excess Water treatment. The intensity of root colonization by *M. robertsii* was weakly associated with the Deficit Water treatment (fig. S2).

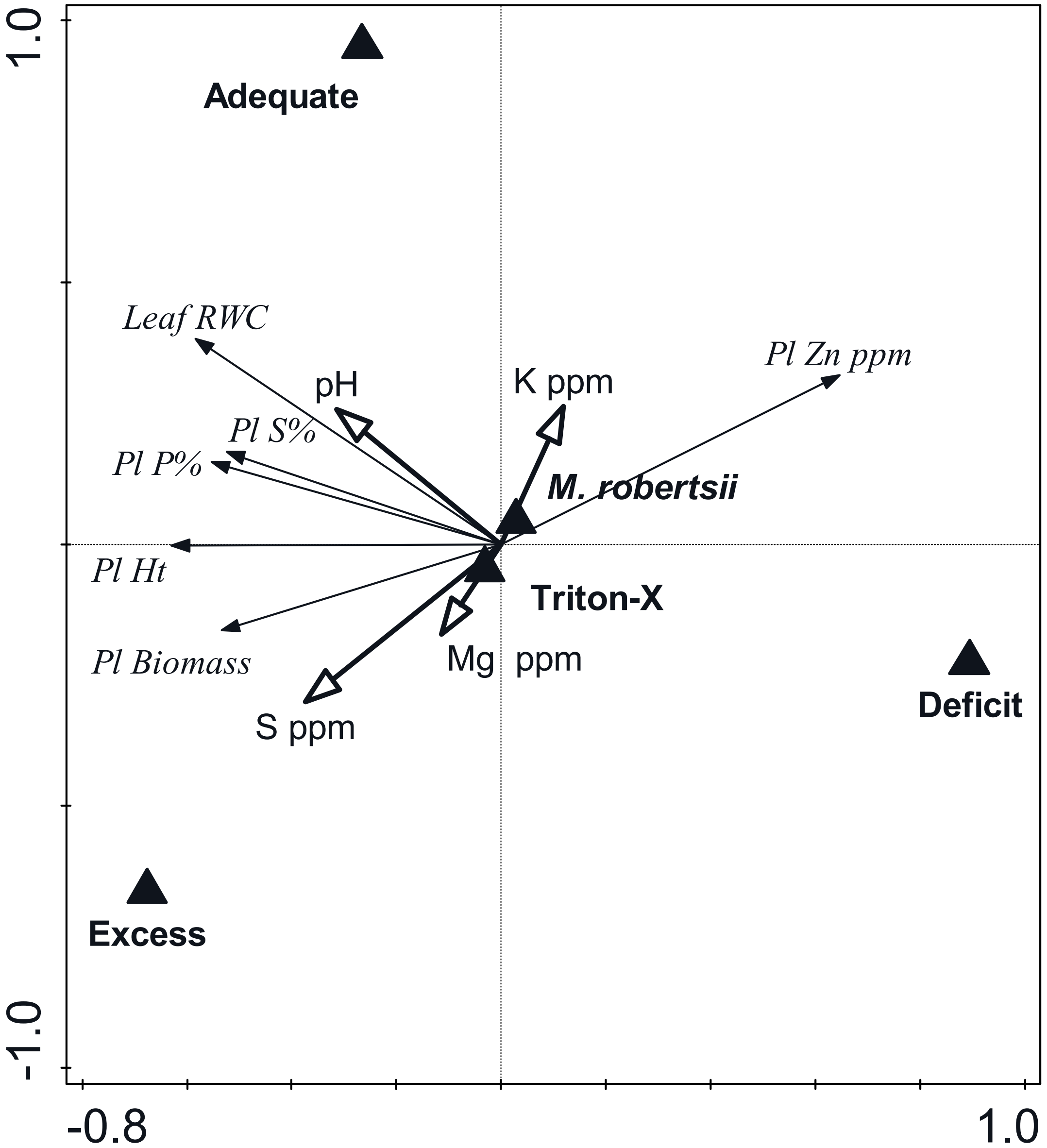

Figure S2. Relationship of plant growth parameters and relative prevalence of *M. robertsii* in the soil at the end of the experiment. Plant growth parameters and relative prevalence of *M. robertsii* in the soil were most closely related to the Excess Water treatment. Water treatment (Axis 1) explained about 16.54% of the explained variation in plant characteristics. Mr treatment (Axis 2) only explained about 0.28% of the variation.

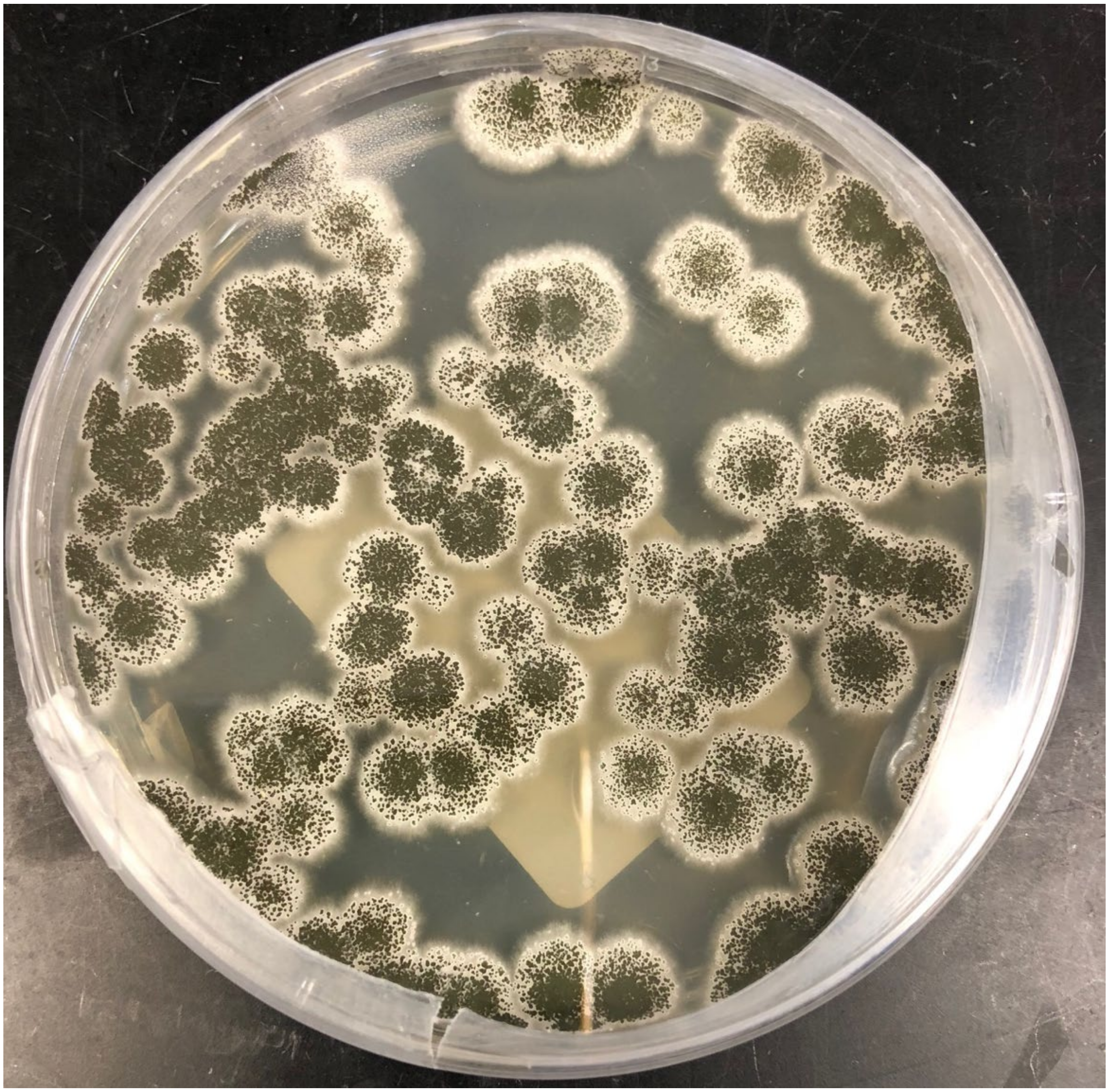

Figure S3. *Metarhizium robertsii* growth on potato dextrose agar (PDA) supplemented with yeast at 25 ± 2 °C for 10 days in darkness. The image shows characteristic dark green conidia with white mycelium.

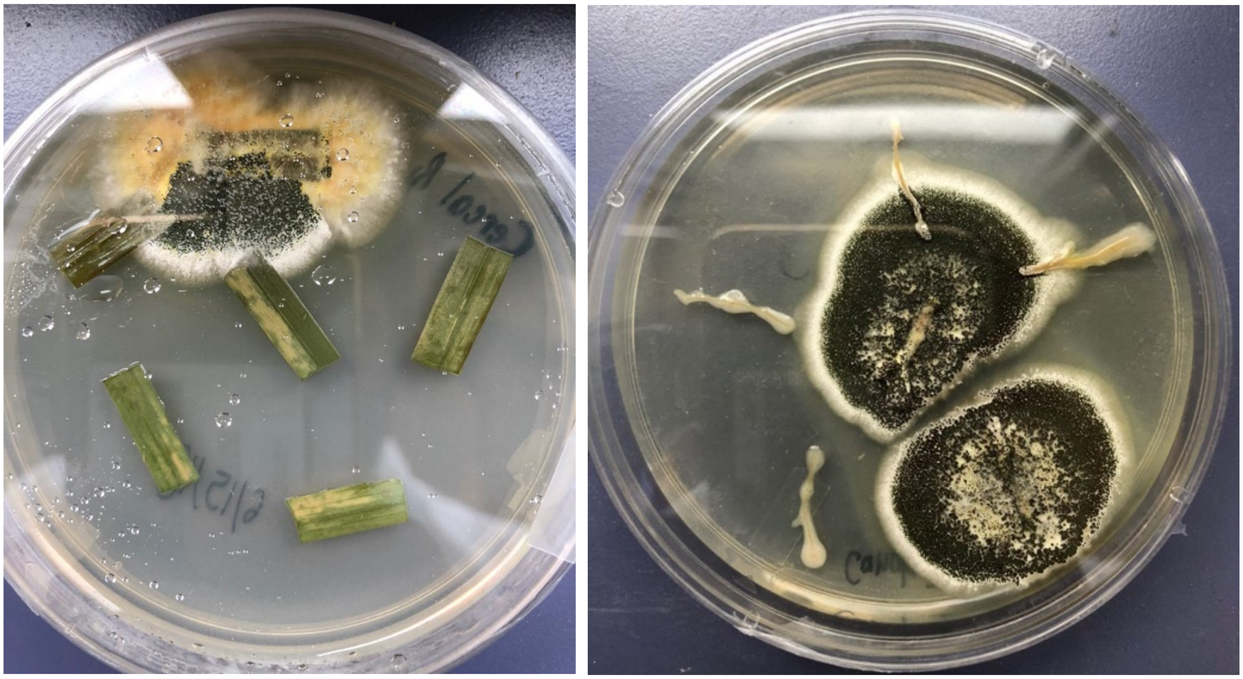

Figure S4. Plant tissues show growth of *M. robertsii* when plated on CTC, a selective medium that confirms endophytic colonization in maize.

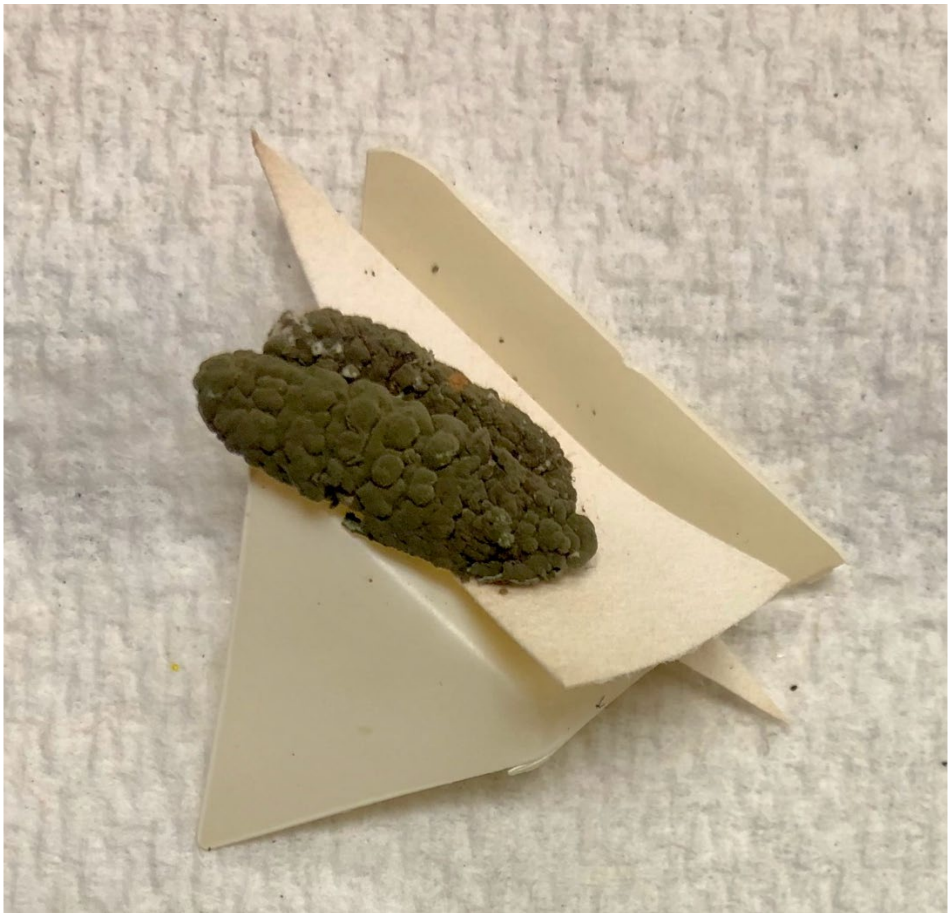

Figure S5. Mealworm larvae covered with dark green conidia of *Metarhizium robertsii.*
